# The effect of long-term care public benefits and insurance on informal care from outside the household: empirical evidence from Italy and Spain

**DOI:** 10.1007/s10198-020-01215-7

**Published:** 2020-07-11

**Authors:** Christophe Courbage, Guillem Montoliu-Montes, Joël Wagner

**Affiliations:** 1 Geneva School of Business Administration, University of Applied Sciences Western Switzerland (HES-SO), Geneva, Switzerland; 2 Department of Actuarial Science, Faculty of Business and Economics (HEC), University of Lausanne, Lausanne, Switzerland; 3 Department of Actuarial Science, Faculty of Business Economics (HEC Lausanne), Swiss Finance Institute, University of Lausanne, Lausanne, Switzerland

**Keywords:** Long-term care, Public benefits, Insurance, Informal care, G22, I11, J14

## Abstract

This article uses cross-sectional data from the Survey of Health, Ageing, and Retirement in Europe (SHARE) database to test the effect of both long-term care (LTC) public benefits and insurance on the receipt of informal care provided by family members living outside the household in Italy and Spain. The choice of Italy and Spain comes from the fact that informal care is rather similar in these two countries while their respective public LTC financing systems are different. Our results support the hypothesis of LTC public support decreasing the receipt of informal care for Spain while reject it for Italy. They tend to confirm that the effect of public benefits on informal care depends on the typology of public coverage for LTC whereby access to proportional benefits negatively influences informal care receipt while access to cash benefits exerts a positive effect. Our results also suggest that private LTC insurance complements the public LTC financing system in place.

## Introduction

The ageing of populations in most industrialised countries is accompanied by an increase in the needs for long-term care^1^ (LTC). Informal caregivers, mainly relatives or family members, and in particular children, meet a large part of LTC needs [1]. Informal care, therefore, contributes to attenuate LTC expenditures’ increases. However, providing informal care could also be detrimental for the caregiver’s physical and mental health and employment participation [2, 3]. Thus, better understanding the determinants of informal care is crucial in designing LTC financing programmes. Several factors, such as the degree of dependency, the number of children, family disintegration, geographical remoteness, women's work, fertility rates and the amount of inheritance, can influence the amount and the organization of informal help [4].

The availability of public and private LTC financing can also influence informal care. In that respect, public LTC support has mainly been shown to decrease informal care. For instance, Ettner [5], Pezzin et al. [6] and Stabile et al. [7], using North American surveys and experimental data, show that increased availability of publicly financed home care is associated with an increase in its utilization and a decline in informal caregiving. However, this hypothesis has been questioned by Motel-Klingebiel et al. [8], who show that the extent to which older people rely on family help is independent of the welfare estate regime in which they live.

The availability of private LTC insurance has also been discussed as potentially reducing informal care. This phenomenon, first introduced by Pauly [9] and labelled intra-family moral hazard, refers to the disincentive of informal caregivers to provide care, because their dependent elderly has insurance coverage against formal LTC costs. It occurs as LTC insurance protects the parent’s bequest from the costs of formal care in case of dependency, thus weakening the child’s main incentive to provide care. Naturally, the same analysis can also be transposed to public LTC benefits as stressed by Zweifel and Strüwe [10].

Various elements need to be taken into account when addressing the effect of LTC coverage on informal care. The first one being the relationship between formal and informal care as addressed by Ettner [5], Pezzin et al. [6] and Stabile et al. [7]. If formal and informal care are substitutes, the availability of subsidised formal care should decrease informal care. Nevertheless, the strength of such substitutability depends on the degree of dependency and on the type of home care considered [11]. Indeed, for severe levels of dependency and high-skilled home care, formal and informal care seem to be complements rather than substitutes [12].

A second element that might drive the effect of LTC financing availability on informal care is the nature of LTC benefits. In that respect, Klimaviciute [13] has theoretically shown that intra-family moral hazard is attenuated when insurance benefits are fixed and not proportional to LTC expenses. The intuition being that with proportional insurance benefits, benefits are received only if formal care is consumed, while fixed benefits do not depend on formal care consumption. Implicitly, proportional benefits protect more the parent’s bequest from the costs of formal care than fixed benefits. The same reasoning could apply to public LTC benefits being either in kind, i.e. rather proportional as received conditionally on the receipt of formal care, or in the form of cash allowances, i.e. rather fixed.

A third element that could influence the link between LTC coverage and informal care is linked to the motives for providing informal care. In particular, apart from the bequest protection motive, informal care can also be provided for altruistic reasons or as a moral obligation [14]. In the case of altruistic caregivers, Courbage and Eeckoudt [15] and Bascans et al. [16] show that more LTC insurance could even increase optimal informal care provision, questioning the existence of a negative effect of insurance on informal care. As for the moral obligation motive, the potential negative effect of insurance on informal care could also be attenuated if caregivers have the feeling they are compelled to take care of their dependent relatives.

The aim of this paper is to investigate empirically the impact of LTC public benefits and LTC insurance ownership on the receipt of informal care by dependent individuals. This article looks at this issue in Italy and Spain using data from the Survey of Health, Ageing, and Retirement in Europe (SHARE) database which deals with the health, lifestyle and financial situation of individuals aged 50 and over in the majority of European countries. We restrict our analysis to care provided by family members living outside the household, excluding help provided by co-resident relatives and other caregivers such as neighbours and friends, which has been shown to be much less sensitive to public and private support [17, 18]. Thus, even if omitted caregivers represent a significant source of informal care, their inclusion into the analysis would come at the cost of a large increase in our estimates’ heterogeneity.

The choice of Italy and Spain stems from the fact that informal care is rather similar in these two countries while their respective public LTC financing systems are rather different. Indeed, sociologists such as Reher [19] suggest a division between Southern Europe “strong family ties” and Northern Europe “weak family ties” countries. The moral obligation motive for caregiving is central in “strong family ties” countries, given that in Southern Europe and in Latin-speaking communities, as opposed to Northern European countries, much of the help given to dependent people is expected to come from the family [20, 21]. However, the nature of public LTC benefits is different in the two countries, proportional to LTC expenses in Spain and mainly in form of cash benefits in Italy. Hence, by studying two relatively similar countries in terms of values and family ties, cultural heterogeneity tends to disappear, allowing us to focus on whether the typology of LTC coverage plays a role in influencing the receipt of informal care. In addition, according to Motel-Klingeibel et al. [8], another source of heterogeneity between welfare regimes could come from differences in the development of welfare services. By selecting Italy and Spain, which have similar ratios of LTC public spending as a percentage of GDP [22], we aim to control also for this second source of heterogeneity.

Our results show that in Spain individuals having access to public LTC benefits are less likely to receive informal care by non-co-resident relatives than those individuals who do not benefit from public LTC support. The opposite is found for Italy. Our findings tend to confirm that the effect of public benefits on informal care depends on the typology of public coverage for LTC whereby access to proportional benefits negatively influences informal care receipt while access to cash benefits exerts a positive effect. Our results also show that private LTC insurance is positively related to the receipt of informal care provided by family members living outside the household in Italy and negatively in Spain even if these results are not always significant at the usual confidence levels. Hence, private LTC insurance seems to complement the public LTC financing system in place, explaining why the direction of the marginal effect of private LTC insurance on informal care follows the one of public LTC support. Such results can be highly relevant in terms of LTC financing policies.

The article is organised as follows. In Section “LTC financing in Italy and Spain”, we present briefly the ways LTC financing is organised in Italy and Spain. Section “Available data and variables” describes the database and the variables used. The econometric analysis and the results are presented in Section “Econometric analysis”. Section “Robustness” consists of robustness checks of the results of Section “Econometric analysis”. The final section offers a conclusion.

## LTC financing in Italy and Spain

### LTC risk coverage in Italy

In Italy, the cost and design of public LTC-related services is highly fragmented and is shared simultaneously between the State, the regions, and the municipalities [23]. Cash benefits are the most important pillar of the Italian LTC public intervention in terms of expenditure and number of older people affected [23]. The main cash benefit, established by the law of 11 February 1980 [24], is the *indennità di accompagnamento* made available by the Social Security in the whole country to severely disabled people needing the permanent help of a relative to carry out the activities of daily living. There exist also some cash benefits provided by the regions, provinces and municipalities [25]. Public home help for personal care and domestic tasks as well as institutional care are managed by municipalities in coordination with the National Health Service.

Whereas health care services for elderly are free of charge in Italy, public home help for personal care and domestic tasks is means-tested and users can pay up to its full cost. There is a wide variation in the co-payment modalities as they are defined by municipalities [25]. The *indennità di accompagnamento* is universal and not means-tested and was set at €508 per month in 2015 independently of the age and place of residence of the recipient. It was granted to 363,868 individuals at the end of 2015 [26]. This cash benefit, which was initially thought as a measure to support informal caregivers, now serves as well to remunerate private home help, in particular help given by migrant workers [23].

To be declared eligible for the *indennità di accompagnamento*, an individual needs to be assessed by a health commission in a specialized centre or clinic after an appropriate period of observation or hospitalization [27] as 100% disabled and dependent, i.e. in need of continuous assistance or unable to walk without the permanent help of a relative. For other regional and municipal LTC services and cash benefits, eligibility criteria are not homogeneous, and each region has a specific dependency classification system taking into account mainly activities of daily living (ADL) limitations and to a lower extent instrumental activities of daily living (IADL) limitations [25].

Finally, the private LTC insurance market is rather thin in Italy [28]. Some insurance companies offer private LTC coverage, with products consisting of a life annuity in case of permanent or full dependency.

### LTC risk coverage in Spain

In Spain, the regions and the municipalities offer universal LTC public coverage following the 39/2006 Law [29]. The *prestaciones y servicios para la autonomía y la dependencia*, i.e. the dependency benefits and services, are granted to all individuals recognized as dependent regardless of their age, geographical location and financial situation. This subsidy can be either in kind in the form of formal care or financial as a percentage of formal care cost. According to the law, in kind subsidies have priority over the financial ones. In kind formal care can be provided at home, in nursing homes and in so called “day” or “night” centres. Financial subsidies can only be used to purchase formal LTC (if publicly provided LTC is not available) or to purchase specific personal assistance services.^2^ In 2015, 745′720 individuals received the *prestaciones y servicios para la autonomía y la dependencia*, either as in kind or financial subsidies [31].

The system is financed via general taxation and means-tested co-payments of the users. The average co-payment is estimated to be €304, €412 and €662 per month for moderately, severely and major dependent respectively, which represents about 50% of the total cost [32]. The assessment of users’ participation to the total cost of LTC is complex due to regional heterogeneity [32, 33].

Severity of dependency is evaluated by a socio-medical team following a visit and an interview at the place of residence of the person applying for public benefits. The evaluation tool is a unified scale that has been approved in 2011 by the Spanish government under the Royal Decree 174/2011 [34]. To be eligible to public services and subsidies, an individual has to be declared at least as moderately dependent, i.e. needing help to perform several ADL at least once a day or needing limited or not continuous help to be autonomous. Initially, only individuals recognised with major and severe dependency were covered by the public LTC system and it was not until mid-2015 that moderately dependent individuals became eligible to public coverage [31].

Finally, in Spain, the private LTC insurance market is rather small with 37,225 insured in 2015 [35]. However, the market is quite dynamic and shows high growth rates. Between 2012 and 2015, the number of insured experienced a growth rate of 29%, probably due to a low starting point [36]. Private insurance benefits can be either in the form of a pre-determined lump-sum or in the form of an annuity. Their eligibility criteria are tighter than the public ones, as private companies only recognize severe dependency corresponding to individuals needing a very high or permanent amount of support to stay autonomous.

We summarize the main characteristics of the Italian and Spanish LTC financing systems in Table 1.

**Table 1:** Summary of the Italian and Spanish LTC financing systems

	Italy		Spain
Public LTC organization	LTC benefits offered by the State, regions and municipalities		Public LTC benefits offered and managed by regions and municipalities
	*Indennità d’accompagnamento* (the most important benefit) provided by the State		Law 39/2006 unifies the basic aspects of the public LTC financing system
Eligibility	*Indennità* is granted to severely disabled, regardless of age		All those recognized at least as moderately dependent, regardless of age
Typology of benefits	Cash benefits mainly (such as the *indennità d’accompagnamento*)		In kind at home or in an institution Financial subsidies to formal care Financial subsidies to at home informal care (if formal care is not available)
Financing	*Indennità* is financed by Social Security		General taxation
	No co-payments		Means-tested co-payments
Private insurance	Life annuity granted to permanent or full dependent		Lump-sum payment or life annuity granted to severely dependent

## Available data and variables

### Data

We use the SHARE database to empirically study the effect of both public support and private LTC insurance ownership on the receipt of informal care in both Italy and Spain. SHARE is a multidisciplinary, longitudinal and cross-national micro-database containing information on health-related variables, labour market variables, economic variables and other variables (including education, housing, social support and family structure) of a representative sample of European individuals aged 50 years or older and their spouses. The first wave of SHARE was released in 2004. SHARE follows the design of the U.S. Health and Retirement study and the English Longitudinal Study of Ageing. For more details on the survey, readers should refer to Börsch-Supan and Jürges [37].

For the purpose of our study, we use data from the sixth wave of the SHARE database. The fieldwork of the sixth wave was completed in 2015, released in 2017 and contains information about 68,231 individuals from 18 different European countries. We discard the use of data from other waves as the Spanish public LTC system was not fully in place until mid-2015.

The subset of SHARE regarding Italy and Spain contains 10,949 observations, 5313 corresponding to Italy and 5636 to Spain. A restriction to individuals having at least one mobility, ADL or IADL limitation leaves us with 5097 observations, 2417 from Italy and 2680 from Spain. In addition, due to missing values for some variables, 236 and 336 observations are lost in the Italian and Spanish samples, respectively (19, respectively, 10 are lost for missing information on limitations). Thus, our final sample includes 4525 observations, 2181 corresponding to Italy and 2344 to Spain. Finally, for models including the control variables *Net wealth* and *Regional dummies*, additional missing values leave us with a total of 3760 and 3932 observations, respectively.

### The variables

In this section, we present the variables used in our analysis, in particular, informal care receipt and LTC coverage, along with their descriptive statistics.

#### Informal care receipt

In SHARE, individuals are asked if any family member, friend or neighbour from outside or inside their household gave help to them and from whom they were given care. Additionally, respondents receiving care from outside the household can indicate what type of help they received, and more specifically, whether the help received was in the form of personal care (e.g., dressing, bathing, getting out of bed), practical household help (e.g., home repairs, transportation, shopping), or help with paper work (e.g., filling out forms, setting financial or legal matters). Interviewed individuals are allowed to declare having received any combination of these three types of help simultaneously.

Based on these answers, we generate three categories of informal care which are informal care in general (simply denoted informal care), informal care for ADL and informal care for IADL, as shown in Table 2. The first category of informal care includes those individuals declaring that they received at least one type of help amongst help with personal care, practical household help, and help with paperwork. In informal care for ADL, we include those individuals declaring having received help with personal care. The informal care for IADL group encompasses those declaring having received practical household help or help with paperwork.

**Table 2:** Informal care from outside the household by country

	Number of observations		% of *N*		
	Italy	Spain	Italy (%)	Spain (%)	Difference (%)
Size of the sample (*N*)	$2181$	$2344$	$100$	$100$	–
Receipt of help					
Total	$580$	$638$	$26.59$	$27.22$	$- 0.63$
Outside the household	$451$	$449$	$20.68$	$19.16$	${ }1.52$
Inside the household	$$185$$	$$287$$	$$8.48$$	$$12.24$$	$$- 3.76^{{***}}$$
Caregiver’s identity					
Family member, outside household	$347$	$402$	$15.91$	$17.15$	$- 1.24$
Other, outside household	$$104$$	$$47$$	$${ }4.77$$	$${ }2.01$$	$$2.76^{***}$$
Family member, inside household	$$177$$	$$269$$	$$8.12$$	$$11.48$$	$${ } - 3.36^{***}$$
Other, inside household	$8$	$18$	${ }0.37$	${ }0.77$	$- 0.40$
Informal care from outside the household by type and caregiver					
Informal care for IADL	$424$	$416$	$19.44$	$17.75$	$1.69$
Family member	$325$	$372$	$14.90$	$15.87$	$- 0.97$
Other	$$99$$	$$44$$	$$4.54$$	$${ }1.88$$	$$2.66^{***}$$
Informal care for ADL	$163$	$209$	$7.48$	$8.91$	$- 1.43^{*}$
Family member	$$138$$	$$193$$	$${ }6.33$$	$$8.23$$	$$- 1.90^{**}$$
Other	${ }25$	$16$	${ }1.15$	${ }0.68$	$0.47$

The significance levels of the two-tailed Welch’s *t* test for difference in means are coded as follows: *significance at 10% level, **significance at 5% level, ***significance at 1% level

Table 2 summarizes for both Italy and Spain whether individuals receive help or not in our sample, the identity of their main caregiver and the type of care they receive by individuals living outside their household.

In both countries, around 27% of the interviewed declare to receive informal care. In Italy, 21% of the sample declares to receive informal care from outside the household and an 8.5% from inside. In Spain, these rates represent a 19% and a 12% of the sample, respectively. Some individuals receive simultaneously both types of care in our sample. Indeed, the sum of those respondents receiving informal care from outside and inside the household exceeds in both countries the number of individuals receiving informal care in general.

Family members from outside the household play a dominant role in providing care, supplying around 60% of total informal care in both countries (347 observations from 580 in Italy and 402 from 638 in Spain). Additionally, in Italy and Spain, more than 90% of those respondents who receive help from outside the household receive it as care for IADL (i.e. 424 respondents over 451 in Italy and 416 over 449 in Spain). Nevertheless, informal care for ADL plays also an important role, representing 36% (163 over 451) of the total amount of care received from non-co-resident in Italy and 47% (209 over 449) in Spain. A substantial number of individuals declare receiving both, help with ADL and IADL simultaneously. Concerning informal care provided by co-resident caregivers, we do not know, unfortunately, exactly its type, even if from the phrasing of the question identifying these caregivers in SHARE we can think that they provide help with ADL only or both types of help simultaneously.^3^

In both countries, help with ADL from outside the household and care from inside the household is almost exclusively provided by family members. Neighbours and friends provide, mainly, only care for IADL and thus, seem to support a lower caregiving burden. From Table 2, we also see that despite the existence of important differences between the Italian and Spanish public LTC financing systems, the differences between both samples concerning informal care are rather weak. The main differences, significant at the 1% level, concern caregiving by non-family members, which is significantly more present in Italy, and caregiving from inside the household, which is more common in Spain.

In the econometric analysis, we examine the effect of public LTC benefits and insurance on informal care receipt provided by relatives living outside the household. Our dependent variable accounts, thus, for around 60% of all informal care received by respondents. Help received by co-resident relatives and other caregivers is excluded and categorized as a zero, since pooling all informal care in a single item would result in a highly heterogeneous dependent variable. This occurs on two grounds. First, because it seems reasonable to expect that informal care provided inside the household, mainly by spouses, is much less sensitive to public and private LTC coverage than other forms of informal care [17]. Second, because compared to relatives, neighbours and friends perform other tasks, have different motives to provide care and their role seems to be complementary to that of spouses and children [18].

We also decided to treat informal care for ADL and IADL as two separate dependent variables in the econometric analysis. The reason is that they could be provided for different reasons. As shown by Bonsang [12], Van Houtven and Norton [38] and Bolin [11], informal care is rather a substitute of less intensive formal care such as help with IADL, but can be a complement to more intensive care such as personal home care. Thus, both types of care could be influenced to a different extent by public LTC support and insurance.

#### LTC coverage

In the survey, individuals are further asked to declare if they own public, private voluntary or private mandatory LTC insurance, or no coverage at all. Public LTC insurance corresponds to insurance or financing provided by the State. Despite the terminology in SHARE, public LTC financing in Italy and Spain does not correspond strictly to a public LTC insurance scheme but to public benefits as indicated in Section “LTC financing in Italy and Spain”. Private mandatory LTC insurance corresponds mainly to private group insurance provided through the employer while private voluntary LTC insurance corresponds to voluntary supplementary or complementary individual insurance. Table 3 reports how individuals in our sample are covered for LTC-related expenses.

**Table 3:** LTC coverage by country

	Number of observations		% of the total *N*		
	Italy	Spain	Italy (%)	Spain (%)	Difference (%)
Size of the sample (*N*)	$2181$	$2344$	$100$	$100$	–
LTC coverage					
Does not own LTC coverage	$$1834$$	$$1648$$	$$84.09$$	$$71.84$$	$${ }12.25^{{***}}$$
Owns LTC coverage	$$347$$	$$660$$	$$15.91$$	$$28.16$$	$$12.25^{{***}}$$
Type of LTC coverage					
Public	$$313$$	$$596$$	$$14.35$$	$$25.43$$	$$- 11.08^{{***}}$$
Private mandatory insurance	$$6$$	$$20$$	$$0.28$$	$$0.85$$	$$- 0.58^{{***}}$$
Private voluntary insurance	$$31$$	$$65$$	$$1.42$$	$$2.77$$	$$- 1.35^{{***}}$$

The significance levels of the two-tailed Welch’s t-test for difference in means are coded as follows: *significance at 10% level, **significance at 5% level, ***significance at 1% level

With regard to the types of LTC coverage, we note that the sum of public, private voluntary and private mandatory coverage can exceed the total number of observations of those owning LTC coverage. This arises as the same individual can have multiple types of coverage at the same time, e.g. public benefits and private voluntary insurance.

In both countries, LTC coverage is mainly provided by the State. In Italy, 14% of the respondents in our sample report having public LTC coverage, and very few report being covered with private voluntary or mandatory LTC insurance. In Spain, the proportion of those receiving public LTC benefits is higher than in Italy with around 25% of the sample declaring being covered by the public system while the number of individuals owning private LTC insurance is much lower and represents around 3% of the total.

One explanation of such discrepancy in public LTC coverage between the two countries could come from eligibility criteria. In Italy, the main LTC benefit (i.e. the *indennità d’accompagnamento*) is only attributed to severely dependent individuals, while in Spain, eligibility to public LTC benefits also includes moderately dependent individuals. The large share of individuals not having any financial LTC coverage in both countries could be explained, in addition to eligibility criteria, by the belief that care should be exclusively a matter of the family, by insufficient information about public LTC programs, by the complexity of the application process to public LTC or by the presence of co-payments, among others.

#### Other variables

In the econometric analysis, we control the effect of public LTC coverage and LTC insurance on informal care receipt with a series of additional variables.

First, we consider the effect of formal home care utilisation on informal care. We use formal care utilisation as a control variable, because our objective is to investigate whether public benefits and private insurance, and not the receipt of formal care, provides incentives or disincentives to informal care. Additionally, formal care availability, which can be proxied by formal care use, could be simultaneously correlated with public LTC support take-up, private LTC insurance ownership and informal care. We define the variable formal care as indicating if the individual received home help with personal care (e.g. dressing, eating or using the toilet), domestic tasks (e.g. cleaning, ironing, cooking, meals-on-wheels) or other activities such as filling a drug dispenser by paid professional workers during the previous 12 months. Following Bolin et al. [11], we also consider highly qualified health care in the form of a binary variable indicating whether the respondent has been in a hospital overnight during the last year and on the number of the interviewee’s visits to a doctor during the previous 12 months. We separately treat formal home care and health care as their relationship with informal care might be different according to the literature [11].

The respondent’s degree of dependency is included as a control since it is the most important driver of informal care provision according to the literature [18]. Following Courbage and Roudaut [39], the level of dependency can be defined through the self-reported number of limitations the individual has with a set of movements (walking 100 m, sitting for 2 h, etc.), ADL (dressing, using the toilet, bathing, etc.) and IADL (phoning, using a map, taking medicines, etc.). The respondent’s self-reported health is also considered, since it can also be an important determinant of informal care besides its positive correlation with the severity of dependency.

As the family structure is very likely to simultaneously affect the supply of informal care and the decision to purchase voluntary LTC insurance [38, 40], we consider a large set of controls describing the respondent’s household and family composition. We include the number of members living in the respondent’s household and his/her number of children, as well as a set of binary variables such as being married, widow, having a co-resident child, and having a daughter.

We include three classical demographic controls, i.e. the respondent’s gender, age, and whether he/she lives in an urban area or not. Finally, we also include net wealth (including housing assets) and a binary variable for whether the interviewee has given a material or financial gift larger than 250€. This is done to control for a possible omitted variable bias as wealth and financial gifts are likely to be simultaneously correlated with informal care (i.e. if bequest or exchange motives for providing care are present), public LTC benefits eligibility (via means-tested co-payments) and private LTC insurance purchase. We do not to include income, education and employment situation as controls since most individuals of the sample have left the labour market. Lastly, we include for Spain a binary variable indicating if the interview was performed in Catalan as cultural and institutional differences between the Catalan-speaking population and the rest of Spaniards, simultaneously affecting informal care receipt and LTC insurance ownership, are likely to be present in our dataset.

### Descriptive statistics

Table 4 provides a summary and description of the set of variables considered in the econometric models. Sample mean values are reported separately for Italy and Spain.

**Table 4:** Variables’ description and sample means

Variable	Description	Italy	Spain	Difference
Dependent variables				
Informal care	1 if having received at least one type of help amongst help with personal care, practical household help, and help with paperwork by a family member from outside the household	$0.159$	$0.172$	$- 0.013$
Informal care for ADL	1 if having received help with personal care (dressing, eating, using the toilet…) by a family member from outside the household	$$0.063$$	$$0.082$$	$${ } - 0.019^{{**}}$$
Informal care for IADL	1 if having received practical household help (gardening, shopping…) or help with paperwork such as filling out forms by a family member from outside the household	$0.149$	$0.159$	$- 0.010$
Independent variables				
LTCI public	1 if reporting to own LTC insurance or financing provided by the State	$$0.144$$	$$0.254$$	$${ } - 0.110^{{***}}$$
LTCI private	1 if reporting to own private mandatory or voluntary / supplementary LTC insurance	$$0.017$$	$$0.036$$	$${ } - 0.019^{{***}}$$
Formal care	1 if having received professional or paid personal care, help with domestic tasks or help with other activities such as filling a drug dispenser during the last year	$$0.116$$	$$0.172$$	$$- 0.056^{{***}}$$
Hospital	1 if having been in a hospital overnight during the last 12 months	$${ }0.177$$	$$0.202$$	$$- 0.025^{{**}}$$
Doctor	Number of doctor visits during the last year	$$11.950$$	$$8.337$$	$${ }3.613^{{***}}$$
Mobility limitations	Number of mobility limitations (walking 100 m, sitting for 2 h, climbing stairs…)	$${ }3.364$$	$$3.958$$	$$- 0.594^{{***}}$$
ADL limitations	Number of limitations in Activities of Daily living (getting in / out of bed, bathing or showering…)	$${ }0.529$$	$$0.682$$	$$- 0.153^{{***}}$$
IADL limitations	Number of limitations in Instrumental Activities of Daily Living (phoning, personal laundry…)	$${ }1.059$$	$$1.529$$	$${ } - 0.470^{{***}}$$
Health	Respondent’s self-reported health	$${ }2.214$$	$$2.103$$	$${ }0.111^{{**}}$$
Household members	Number of people living in the respondent’s household, excluding lodgers	$2.263$	$2.224$	${ }0.039$
Married	1 if reporting to be married or in a registered partnership	$0.644$	$0.626$	${ }0.018$
Widow	1 if reporting to be widow	$${ }0.165$$	$$0.191$$	$${ } - 0.026^{{**}}$$
*N* children	Interviewee’s number of living children	$${ }1.997$$	$$2.264$$	$${ } - 0.267^{{***}}$$
Co-resident children	1 if reporting to have a child living in the same household	$${ }0.251$$	$$0.190$$	$${ }0.061^{{***}}$$
Has daughter	1 if reporting to have at least one living daughter	$${ }0.630$$	$$0.668$$	$${ } - 0.038^{{***}}$$
Care other	1 if having received informal care by a neighbour or a friend from outside the household	$${ }0.048$$	$$0.020$$	$${ }0.028^{{***}}$$
Care inside	1 if having received informal care by somebody from inside the household	$${ }0.085$$	$$0.122$$	$${ } - 0.037^{{***}}$$
Age	Interviewee’s age	$$70.712$$	$$74.331$$	$$- 3.619^{***}$$
Female	1 if the interviewee is a woman	${ }0.628$	$0.613$	${ }0.015$
Urban	1 if the interviewee lives in a big city, the suburbs or outskirts of a big city or a small town	$${ }0.341$$	$$0.419$$	$${ } - 0.078^{***}$$
Net wealth (in €)	Self-reported net wealth, in euro	$200,423$	$205,396$	${ } - 4972$
Gift	1 if the interviewee has given any material or financial gift of 250€ or more in the last 12 months	$${ }0.296$$	$$0.096$$	$${ }0.200^{{***}}$$
Catalan	1 if interviewed in Catalan (Spain)	–	$0.167$	–

Number of observations: 2181 in Italy and 2344 in Spain (with the exception of the variable Net wealth)

The significance levels of the Welch two-tailed *t* test for difference in means are coded as: *significance at 10% level, **significance at 5% level, ***significance at 1% level

There is a significant overlap on the dependent variables *Informal care* and *Informal care for IADL,* as more than 90% of those who receive help by family members living outside the household receive it as care for IADL. While SHARE distinguishes private mandatory and voluntary LTC insurance, we merge them into one type of private insurance (variable LTCI private) to work with a variable maximizing the number of individuals privately covered. The informal care and insurance related variables’ sample means reflect the trends commented earlier in Sections “Informal care receipt” and “LTC coverage”. Additionally, Italians are less likely to receive formal home care when compared to Spaniards which is consistent with the observed differences in public LTC coverage. In both countries the average household is composed of 2.2 members and surveyed individuals have on average about 2 children. Roughly 63% of the individuals are married, around 18% are widow and between 20 and 25% of respondents live with their children.

## Econometric analysis

### Econometric specification

In our econometric analysis, we run three probit models on the three binary variables defining informal care provided by family members from outside the household (see Table 4). This type of regression is suited when the dependent variable takes only two values. More formally, for each country, we model an individual’s probability of receiving informal care by the following equation:

1 $${\text{IC}}_{i}^{j} = \alpha + \beta_{1}^{j} {\text{LTC}}_{i} + \beta_{2}^{j} X_{i} + \varepsilon_{i} ,$$

where ${\text{IC}}_{i}^{j}$ with *j* = 1, 2, 3 corresponds to the three dummy variables defining informal care in an aggregate way (*j* = 1), for ADL (*j* = 2) and for IADL (*j* = 3). While the superscript *j* is linked to the three regressions, the subscript *i* is linked to the observations, i.e. the responses from the surveyed individuals. ${\text{LTC}}_{i}$ refers to the two variables defining public and private LTC financing and $X_{i}$ to the independent variables in Table 4 selected as control variables. Assuming the error term $\varepsilon_{i}$ is normally distributed with mean zero, Eq. (1) can be estimated using a probit model.

We consider as control variables for the final model only those variables in Table 4 which fulfil two criteria. The first is to decrease the Akaike information criterion (AIC) [41] and the second is to be statistically significant at the 10% level. These criteria ensure that the selected variables improve the model’s goodness of fit without raising substantially the risk of overfitting. For the degree of dependency, only the variable with the highest explanatory power among the three mobility, ADL and IADL limitations is included, because of the large collinearity existing between them (i.e. *ρ* > 0.6). The selection of covariates is performed in the joint regression model with IC^1^ as dependent variable (first column of Table 6). We retain this selection across the other models to have a unified specification.

Controls were tested under different forms (linear, binary and categorical) and we retained the one improving the most the AIC. Alternative specifications including more controls were also tested for all regressions. Results did not change substantially, validating our method for the selection of covariates. Finally, we also investigate the relationship between LTC financing and the probability of formal care use. In this case, our dependent variable is the formal home care utilization variable and LTC financing, informal care receipt by family members and the selected controls are used as explanatory variables.

### Empirical results

The numerical results from the model calibration are presented in Table 5.

**Table 5:** Empirical results by type of care and by country

		Informal care (${\text{IC}}^{1}$)		Informal care ADL (${\text{IC}}^{2}$)		Informal care IADL (${\text{IC}}^{3}$)		Informal care (${\text{IC}}^{1}$)		Formal care (${\text{FC}}$)	
		Italy	Spain	Italy	Spain	Italy	Spain	Italy	Spain	Italy	Spain
(Intercept)		$$- 1.994^{{***}}$$ $$\left( {0.350} \right)$$	$$- 1.280^{{***}}$$ $$\left( {0.341} \right)$$	$$- 2.706^{{***}}$$ $$\left( {0.485} \right)$$	$$- 1.871^{{***}}$$ $$\left( {0.443} \right)$$	$$- 2.048^{{***}}$$ $$\left( {0.357} \right)$$	$$- 1.479^{{***}}$$ $$\left( {0.346} \right)$$	$$- 1.895^{{***}}$$ $$\left( {0.398} \right)$$	$$- 0.977^{{**}}$$ $$\left( {0.383} \right)$$	$$- 2.317^{{***}}$$ $$\left( {0.388} \right)$$	$$- 2.687^{{***}}$$ $$\left( {0.340} \right)$$
LTCI public		$$0.233^{{**}}$$ $$\left( {0.093} \right)$$	$$- 0.434^{{***}}$$ $$\left( {0.085} \right)$$	$${ }0.294^{{**}}$$ $$\left( {0.124} \right)$$	$$- 0.214^{{**}}$$ $$\left( {0.103} \right)$$	$${ }0.258^{{***}}$$ $$\left( {0.094} \right)$$	$$- 0.435^{{***}}$$ $$\left( {0.086} \right)$$	$$0.227^{{**}}$$ $$\left( {0.099} \right)$$	$$- 0.478^{{***}}$$ $$\left( {0.095} \right)$$	$$0.244^{{**}}$$ $$\left( {0.102} \right)$$	$${ }0.150^{*}$$ $$\left( {0.077} \right)$$
LTCI private		$$0.413$$ $$\left( {0.268} \right)$$	$$- 0.339{ }$$ $$\left( {0.226} \right)$$	$$0.187$$ $$\left( {0.456} \right)$$	$$- 0.104$$ $$\left( {0.275} \right)$$	$$0.477^{*}$$ $$\left( {0.269} \right)$$	$$- 0.402^{*}$$ $$\left( {0.239} \right)$$	$$0.425$$ $$\left( {0.293} \right)$$	$$- 0.272$$ $$\left( {0.248} \right)$$	$$0.530^{*}$$ $$\left( {0.273} \right)$$	$${ }0.386^{{**}}$$ $$\left( {0.180} \right)$$
Formal care		$$0.084$$ $$\left( {0.101} \right)$$	$$0.370^{***}$$ $$\left( {0.087} \right)$$	$$- 0.005$$ $$\left( {0.129} \right)$$	$$0.240^{**}$$ $$\left( {0.104} \right)$$	$$0.082$$ $$\left( {0.103} \right)$$	$$0.310^{***}$$ $$\left( {0.087} \right)$$	$$0.105$$ $$\left( {0.110} \right)$$	$$0.394^{***}$$ $$\left( {0.097} \right)$$	–	–
Informal care		–	–	–	–	–	–	–	–	$$0.073$$ $$\left( {0.099} \right)$$	$$0.357^{***}$$ $$\left( {0.087} \right)$$
Hospital		$$0.129$$ $$\left( {0.002} \right)$$	$$0.208^{***}$$ $$\left( {0.082} \right)$$	$$0.194^{*}$$ $$\left( {0.115} \right)$$	$$0.284^{***}$$ $$\left( {0.098} \right)$$	$$0.100$$ $$\left( {0.091} \right)$$	$$0.182^{**}$$ $$\left( {0.083} \right)$$	$$0.141$$ $$\left( {0.097} \right)$$	$$0.218^{**}$$ $$\left( {0.091} \right)$$	$$0.251^{***}$$ $$\left( {0.095} \right)$$	$$0.084$$ $$\left( {0.083} \right)$$
IADL limitations		$$0.073^{***}$$ $$\left( {0.016} \right)$$	$$0.108^{***}$$ $$\left( {0.014} \right)$$	$$0.135^{***}$$ $$\left( {0.019} \right)$$	$$0.149^{***}$$ $$\left( {0.017} \right)$$	$$0.067^{***}$$ $$\left( {0.016} \right)$$	$$0.108^{***}$$ $$\left( {0.015} \right)$$	$$0.068^{***}$$ $$\left( {0.018} \right)$$	$$0.111^{***}$$ $$\left( {0.016} \right)$$	$$0.086^{***}$$ $$\left( {0.016} \right)$$	$$0.123^{***}$$ $$\left( {0.014} \right)$$
Health		$$- 0.112^{**}$$ $$\left( {0.046} \right)$$	$$- 0.242^{***}$$ $$\left( {0.048} \right)$$	$$- 0.135^{**}$$ $$\left( {0.084} \right)$$	$$- 0.280^{***}$$ $$\left( {0.065} \right)$$	$$- 0.118^{**}$$ $$\left( {0.047} \right)$$	$$- 0.214^{***}$$ $$\left( {0.048} \right)$$	$$- 0.134^{***}$$ $$\left( {0.050} \right)$$	$$- 0.298^{***}$$ $$\left( {0.053} \right)$$	$$- 0.158^{***}$$ $$\left( {0.053} \right)$$	$$- 0.107^{***}$$ $$\left( {0.046} \right)$$
HH members											
2		$$- 0.476^{***}$$ $$\left( {0.087} \right)$$	$${ } - 0.377^{***}$$ $$\left( {0.088} \right)$$	$$0.432^{***}$$ $$\left( {0.118} \right)$$	$$- 0.211^{*}$$ $$\left( {0.113} \right)$$	$$- 0.493^{***}$$ $$\left( {0.088} \right)$$	$$- 0.396^{***}$$ $$\left( {0.089} \right)$$	$$- 0.382^{***}$$ $$\left( {0.096} \right)$$	$$- 0.406^{***}$$ $$\left( {0.098} \right)$$	$$- 0.499^{***}$$ $$\left( {0.094} \right)$$	$$- 0.256^{***}$$ $$\left( {0.086} \right)$$
3		$$- 0.546^{***}$$ $$\left( {0.119} \right)$$	$${ } - 0.672^{***}$$ $$\left( {0.123} \right)$$	$$0.484^{***}$$ $$\left( {0.164} \right)$$	$$- 0.434^{***}$$ $$\left( {0.159} \right)$$	$$- 0.569^{***}$$ $$\left( {0.122} \right)$$	$$- 0.654^{***}$$ $$\left( {0.125} \right)$$	$$- 0.516^{***}$$ $$\left( {0.135} \right)$$	$$- 0.639^{***}$$ $$\left( {0.134} \right)$$	$$- 0.544^{***}$$ $$\left( {0.133} \right)$$	$$- 0.542^{***}$$ $$\left( {0.122} \right)$$
4 or more		$$- 0.737^{***}$$ $$\left( {0.153} \right)$$	$${ } - 0.782^{***}$$ $$\left( {0.162} \right)$$	$$0.452^{***}$$ $$\left( {0.196} \right)$$	$$- 0.590^{***}$$ $$\left( {0.211} \right)$$	$$- 0.731^{***}$$ $$\left( {0.156} \right)$$	$$- 0.732^{***}$$ $$\left( {0.163} \right)$$	$$- 0.645^{***}$$ $$\left( {0.177} \right)$$	$$- 0.824^{***}$$ $$\left( {0.182} \right)$$	$$- 0.637^{***}$$ $$\left( {0.171} \right)$$	$$- 0.912^{***}$$ $$\left( {0.184} \right)$$
*N* children		$$0.028$$ $$\left( {0.029} \right)$$	$$0.048^{**}$$ $$\left( {0.022} \right)$$	$$0.064^{*}$$ $$\left( {0.037} \right)$$	$$0.023$$ $$\left( {0.028} \right)$$	$$0.032$$ $$\left( {0.029} \right)$$	$$0.034$$$$\left( {0.023} \right)$$	$$- 0.014$$ $$\left( {0.033} \right)$$	$$0.058^{**}$$ $$\left( {0.025} \right)$$	$$- 0.026$$ $$\left( {0.033} \right)$$	$$- 0.026$$ $$\left( {0.024} \right)$$
Has daughter		$$0.158^{*}$$ $$\left( {0.084} \right)$$	$$0.397^{***}$$ $$\left( {0.090} \right)$$	$$0.244^{**}$$ $$\left( {0.120} \right)$$	$$0.375^{***}$$ $$\left( {0.120} \right)$$	$$0.151^{*}$$ $$\left( {0.085} \right)$$	$$0.380^{***}$$ $$\left( {0.092} \right)$$	$$0.246^{***}$$ $$\left( {0.092} \right)$$	$$0.428^{***}$$ $$\left( {0.101} \right)$$	$$- 0.048$$ $$\left( {0.092} \right)$$	$$- 0.034$$ $$\left( {0.086} \right)$$
Age		$$0.018^{***}$$ $$\left( {0.004} \right)$$	$$0.008^{**}$$ $$\left( {0.004} \right)$$	$$0.015^{***}$$ $$\left( {0.006} \right)$$	$$0.006$$$$\left( {0.005} \right)$$	$$0.018^{***}$$ $$\left( {0.004} \right)$$	$$0.010^{***}$$ $$\left( {0.004} \right)$$	$$0.019^{***}$$ $$\left( {0.005} \right)$$	$$0.005$$ $$\left( {0.005} \right)$$	$$0.020^{***}$$ $$\left( {0.005} \right)$$	$$0.023^{***}$$ $$\left( {0.004} \right)$$
Female		$$0.109$$ $$\left( {0.074} \right)$$	$$0.103$$ $$\left( {0.074} \right)$$	$$0.082$$ $$\left( {0.103} \right)$$	$$0.061$$ $$\left( {0.094} \right)$$	$$0.117$$ $$\left( {0.075} \right)$$	$$0.146^{*}$$ $$\left( {0.075} \right)$$	$$0.097$$ $$\left( {0.080} \right)$$	$$0.071$$ $$\left( {0.082} \right)$$	$$0.174^{**}$$ $$\left( {0.083} \right)$$	$$0.149^{**}$$ $$\left( {0.073} \right)$$
Urban		$$- 0.149^{**}$$ $$\left( {0.074} \right)$$	$$- 0.247^{***}$$ $$\left( {0.072} \right)$$	$$- 0.048$$ $$\left( {0.102} \right)$$	$$- 0.143$$ $$\left( {0.092} \right)$$	$$- 0.200^{**}$$ $$\left( {0.076} \right)$$	$$- 0.266^{***}$$ $$\left( {0.074} \right)$$	$$- 0.172^{**}$$ $$\left( {0.082} \right)$$	$$- 0.261^{***}$$ $$\left( {0.079} \right)$$	$$0.188^{**}$$ $$\left( {0.080} \right)$$	$$0.027$$ $$\left( {0.073} \right)$$
Wealth quartiles											
2nd		–	–	–	–	–	–	$$- 0.052$$ $$\left( {0.100} \right)$$	$$0.269^{**}$$ $$\left( {0.109} \right)$$	–	–
3rd		–	–	–	–	–	–	$$- 0.399^{***}$$ $$\left( {0.112} \right)$$	$$- 0.058$$ $$\left( {0.102} \right)$$	–	–
4th		–	–	–	–	–	–	$- 0.178^{*}$ $\left( {0.105} \right)$	$- 0.017$ $\left( {0.010} \right)$	–	–
Catalan		–	$$- 0.527^{***}$$ $$\left( {0.113} \right)$$	–	$$- 0.455^{***}$$ $$\left( {0.156} \right)$$	–	$$- 0.492^{***}$$ $$\left( {0.114} \right)$$	–	$$- 0.378$$ $$\left( {0.137} \right)$$	–	$$0.279^{***}$$ $$\left( {0.095} \right)$$
Pseudo *R*^2a^		11.30%	21.29%	19.35%	26.17%	11.59%	18.67%	12.21%	23.13%	14.93%	19.13%
*N*		2181	2344	2181	2344	2181	2344	1850	1910	2181	2344

Dependent variables are indicated in the first row

Robust standard errors are reported in parentheses

The significance levels of the two-tailed hypothesis test are coded as follows: *significance at 10% level, **significance at 5% level, ***significance at 1% level

^a^ Mc. Fadden’s pseudo *R*^2^

In Italy, public LTC support is found to impact significantly and positively informal care (${\text{IC}}^{1}$) in the first regression. The opposite result is found for Spain, where public LTC coverage has a significant negative impact on informal care provided by family members. This result would tend to confirm our initial hypothesis that different public LTC financing typologies may have a different impact on informal care. On one hand, in Italy, public LTC is characterized by a mixed system consisting of a universal national cash benefit granted to severely dependent (the *indennità d’accompagnamento*), complemented by a very heterogeneous set of additional cash and in-kind benefits provided and regulated at the regional and municipal levels. On the other hand, in Spain, while either in-kind or financial benefits are also granted to moderately dependent, they depend on formal care consumption. Hence, in Spain, benefits are received conditionally on the receipt of formal care which is likely to provide disincentives for informal care. This does not necessarily happen in Italy where cash benefits, the most important pillar of public LTC support, are not conditioned on the receipt of formal care and have more strict eligibility criteria. In Italy, cash benefits can be used to compensate informal caregivers which may explain the positive relationship between public coverage and informal care. Hence, such cash benefits would fulfil their initial role thought to support informal caregivers [23]. These first findings would tend to support the hypothesis of LTC public support decreasing the receipt of informal care for Spain but would reject it for Italy.

Regarding the effect of private LTC insurance on informal care, its effect is positive for Italy and negative for Spain, but not significant at the relevant levels (*p* value of 12.3% in Italy and 13.3% in Spain). Private insurance, whose indemnities take the form of cash benefits, seems to complement the public LTC financing system in place. This could explain why the coefficient corresponding to this variable has the same sign as the one of public LTC coverage. For instance, the negative relationship in Spain could be explained by the fact that private benefits complement the public system and are used to finance co-payments and/or additional formal care costs not fully covered by public benefits.

When informal care is defined only in terms of help with ADL or with IADL (second and third columns in Table 5), our results do not importantly change. When care is defined as informal care for IADL, the main difference is that the effect of private LTC insurance becomes statistically significant at the 10% level in both countries. Additionally, the estimate corresponding to public LTC suffers from an important reduction in Spain when informal care is defined as help with ADL. As help with ADL is a more intense form of care, this result is consistent with the findings of Bonsang [12] and Bolin [11] showing that formal and informal care are weaker substitutes if the intensity of care is high.

In the fourth column, we run a regression with the general definition of informal care as the dependent variable by additionally controlling for the individual’s net assets including housing wealth. More specifically, we include three dummies related to the country-specific quartiles of the sample wealth distribution. When including this variable, the sample size is substantially reduced due to the presence of missing values. Our results show that the coefficients corresponding to public LTC benefits do not suffer relevant changes, whereas those corresponding to private LTC insurance are reduced and become non-significant. Furthermore, we find that wealth has a non-linear effect on informal care.

The fifth set of results contains the regression models using formal care as dependent variable. As laid out above, we control for informal care receipt and for the rest of the variables except wealth (results do not change when we include it). As suspected, in Spain, where public LTC support is conditioned to formal care receipt, we find a positive association between LTC coverage, both public and private, and formal home care. In Italy, where the system is mixed, we find as well public and private LTC coverage to be positively associated with formal home care. Hence, on one hand, in Spain, LTC public benefits increase formal care and decrease informal care. On the other hand, in Italy, LTC public benefits increase both formal and informal care receipt.

Concerning the rest of the control variables, formal care is not significantly correlated with informal care in Italy, while it is significantly positively correlated with the three categories of informal care in Spain. A causal interpretation for this control variable’s marginal effect is beyond the scope of this paper. Indeed, we include formal care utilisation in the regression as an independent variable to control for the effects of public support and private insurance in both countries by formal home care availability. Having been in the hospital is significantly and positively associated with the three categories of informal care as well as with formal care. The number of IADL limitations is positively and significantly related to the receipt of both informal and formal care and a better health has the opposite effect on both variables. The number of members in the respondent’s household has a very significant increasing negative effect on both informal care from outside the household and formal care. The remaining variables proxying co-residential informal care (i.e. the marital status or having a co-resident child or caregiver) are not included as they become non-significant once we control by *Household Members*. Having a daughter, which can be considered as a proxy of informal care supply [12], is significantly positively related to the probability of receiving informal care from outside the household. Age is positively and strongly related with having received both informal and formal care, with the exception of informal care for ADL in Spain (*p* value of 0.102). In most cases, being a woman cannot be significantly related with care receipt. Finally, individuals speaking Catalan are less likely to receive informal care from a family member living outside the household and more likely to receive formal care than the rest of Spaniards.

We also computed the correlation matrix between independent variables and performed variance inflation factor (VIF) checks on all regressions. No major correlations or high values on these tests were found, indicating the absence of major multicollinearity.

### Joint regressions

In this subsection, we check if the differences found between Italy and Spain in the effects of LTC financing on informal care receipt by family members from outside the household are statistically significant in addition of having different signs. To do so, we run the regressions of Table 5 where informal care is the dependent variable without splitting the data in two country samples. We include a country dummy for *Italy* and the interactions LTCI public*Italy and LTCI private*Italy. We remove the dummy variable *Catalan* as we focus on the international differences. The results of this model are displayed in Table 6.

**Table 6:** Empirical results of the joint regression models for informal care

	Informal care (${\text{IC}}^{1}$)	Informal care ADL (${\text{IC}}^{2}$)	Informal care IADL $\left( {{\text{IC}}^{3} } \right)$	Informal care (${\text{IC}}^{1}$)
(Intercept)	$$- 1.554^{{***}}$$ $$\left( {0.244} \right)$$	$$- 3.148^{{***}}$$ $$\left( {0.282} \right)$$	$$- 1.691^{{***}}$$ $$\left( {0.249} \right)$$	$${ } - 1.346^{{***}}$$ $$\left( {0.257} \right)$$
LTCI public	$$- 0.363^{{***}}$$ $$\left( {0.081} \right)$$	$$- 0.178^{*}$$ $$\left( {0.100} \right)$$	$$- 0.370^{{***}}$$ $$\left( {0.083} \right)$$	$$- 0.420^{{***}}$$ $$\left( {0.090} \right)$$
LTCI private	$- 0.315$ $\left( {0.221} \right)$	$- 0.111$ $\left( {0.271} \right)$	$- 0.373$ $\left( {0.235} \right)$	$- 0.242$ $\left( {0.241} \right)$
Formal care	$$0.243^{***}$$ $$\left( {0.065} \right)$$	$$0.140^{*}$$ $$\left( {0.079} \right)$$	$$0.210^{***}$$ $$\left( {0.066} \right)$$	$$0.270^{***}$$ $$\left( {0.072} \right)$$
Hospital	$$0.157^{***}$$ $$\left( {0.060} \right)$$	$$0.235^{***}$$ $$\left( {0.073} \right)$$	$$0.131^{*}$$ $$\left( {0.061} \right)$$	$$0.166^{**}$$ $$\left( {0.065} \right)$$
IADL limitations	$$0.096^{***}$$ $$\left( {0.010} \right)$$	$$0.145^{***}$$ $$\left( {0.012} \right)$$	$$0.094^{***}$$ $$\left( {0.011} \right)$$	$$0.095^{***}$$ $$\left( {0.012} \right)$$
Health	$$- 0.182^{{***}}$$ $$\left( {0.033} \right)$$	$$- 0.214^{{***}}$$ $$\left( {0.046} \right)$$	$$- 0.173^{{***}}$$ $$\left( {0.033} \right)$$	$$- 0.216^{{***}}$$ $$\left( {0.036} \right)$$
HH members				
2	$$- 0.429^{***}$$ $$\left( {0.061} \right)$$	$$- 0.317^{***}$$ $$\left( {0.080} \right)$$	$$- 0.455^{{***}}$$ $$\left( {0.062} \right)$$	$$- 0.414^{{***}}$$ $$\left( {0.067} \right)$$
3	$$- 0.632^{***}$$ $$\left( {0.084} \right)$$	$$- 0.475^{***}$$ $$\left( {0.112} \right)$$	$$- 0.631^{{***}}$$ $$\left( {0.086} \right)$$	$$- 0.611^{{***}}$$ $$\left( {0.093} \right)$$
4 or more	$$- 0.789^{***}$$ $$\left( {0.110} \right)$$	$$- 0.532^{***}$$ $$\left( {0.142} \right)$$	$$- 0.759^{{***}}$$ $$\left( {0.112} \right)$$	$$- 0.791^{{***}}$$ $$\left( {0.125} \right)$$
*N* children	$$0.054^{***}$$ $$\left( {0.017} \right)$$	$$0.046^{**}$$ $$\left( {0.022} \right)$$	$$0.045^{**}$$ $$\left( {0.018} \right)$$	$$0.045^{**}$$ $$\left( {0.019} \right)$$
Has daughter	$$0.256^{***}$$ $$\left( {0.060} \right)$$	$$0.303^{***}$$ $$\left( {0.083} \right)$$	$$0.249^{***}$$ $$\left( {0.062} \right)$$	$$0.308^{***}$$ $$\left( {0.067} \right)$$
Age	$$0.011^{***}$$ $$\left( {0.003} \right)$$	$$0.010^{**}$$ $$\left( {0.004} \right)$$	$$0.013^{***}$$ $$\left( {0.003} \right)$$	$$0.010^{***}$$ $$\left( {0.003} \right)$$
Female	$$0.095^{*}$$ $$\left( {0.052} \right)$$	$$0.068$$ $$\left( {0.069} \right)$$	$$0.122^{**}$$ $$\left( {0.053} \right)$$	$$0.070$$ $$\left( {0.057} \right)$$
Urban	$$- 0.160^{***}$$ $$\left( {0.051} \right)$$	$$- 0.069$$ $$\left( {0.067} \right)$$	$$- 0.196^{***}$$ $$\left( {0.051} \right)$$	$$- 0.204^{***}$$ $$\left( {0.056} \right)$$
Wealth quartiles				
2nd	–	–	–	$0.128^{*}$ $\left( {0.071} \right)$
3rd	–	–	–	$$- 0.203^{***}$$$$\left( {0.075} \right)$$
4th	–	–	–	$- 0.101$ $\left( {0.078} \right)$
Italy	$- 0.024$ $\left( {0.055} \right)$	$- 0.049$ $\left( {0.074} \right)$	$- 0.030$ $\left( {0.056} \right)$	$- 0.043$ $\left( {0.061} \right)$
Italy * LTCI public	$$0.622^{***}$$ $$\left( {0.123} \right)$$	$$0.485^{***}$$ $$\left( {0.160} \right)$$	$$0.653^{***}$$ $$\left( {0.126} \right)$$	$$0.689^{***}$$ $$\left( {0.134} \right)$$
Italy * LTCI private	$$0.785^{**}$$ $$\left( {0.347} \right)$$	$$0.273$$ $$\left( {0.542} \right)$$	$$0.902^{***}$$ $$\left( {0.356} \right)$$	$$0.751^{**}$$ $$\left( {0.377} \right)$$
Pseudo *R*^2a^	15.28%	22.42%	15.08%	16.63%
*N*	4525	4525	4525	3760

Dependent variables in the first row

Robust standard errors are reported in parentheses. The significance levels of the two-tailed hypothesis test are coded as follows: *significance at 10% level, **significance at 5% level, ***significance at 1% level

^a^Mc. Fadden’s pseudo R

The effect of public LTC coverage in Spain is still significant at the 1% level when informal care is defined in general (columns 1 and 4) and as help with IADL (column 3). When informal care is defined as help with ADL, the effect of public LTC coverage is reduced substantially and the negative effect in Spain is only significant at the 10% level, which is consistent with the previous section’s findings, i.e. the effect of public LTC coverage is lower when informal care is defined as help with ADL. Concerning private LTC insurance, the results found previously are maintained as the effect of this variable is negative and not significant in general.

Looking at the country dummy, we find, as in the descriptive statistics, that despite the important differences between the Italian and Spanish public LTC systems, there are virtually no differences in the probability of receiving informal care. The country dummy is not significant in any case.

Considering the interaction LTCI public*Italy, our previously observed differences hold and are even found to be highly significant whatever the definition of informal care used. Concerning the effect of private LTC insurance ownership, differences are significant at the 5% level only when informal care is defined using the general definition and with help for IADL (columns 1, 3 and 4). The differences found previously are then robust and economically relevant given the significance of these interaction terms.

Finally, regarding the other control variables, no substantial changes are observed.

## Robustness

### Controlling for regional fixed effects

As stressed earlier, important heterogeneity at the regional level is present in the Italian and Spanish public LTC systems. In Italy, the main public LTC benefit is granted at the national level but regions and municipalities also fund additional forms of cash and in kind public support. In Spain, public LTC support is regulated at the national level by the law 39/2006 but Autonomous Communities are left with a great deal of discretion for determining co-payment rates and some characteristics of the benefits granted.

To address such heterogeneity, we again run a selection of the regressions of Table 5 and include a set of binary control variables corresponding to the regions in Italy and the Autonomous Communities in Spain. In Table 7, we report the coefficients corresponding to public and private LTC coverage. We do not estimate the model where wealth is included as a control as this alternative specification does not substantially affect the coefficients corresponding to public and private LTC coverage (see Table 5).

**Table 7:** Probit regression models controlling for regional fixed effects

	Informal care (${\text{IC}}^{1}$)		Informal care ADL (${\text{IC}}^{2}$)		Informal care IADL $\left( {{\text{IC}}^{3} } \right)$	
	Italy	Spain	Italy	Spain	Italy	Spain
(Intercept)	$$- 2.020^{{***}}$$ $$\left( {0.405} \right)$$	$${ } - 1.615^{{***}}$$ $$\left( {0.458} \right)$$	$$- 2.270^{{***}}$$ $$\left( {0.562} \right)$$	$$- 2.452^{{***}}$$ $$\left( {0.666} \right)$$	$$- 2.060^{{***}}$$ $$\left( {0.416} \right)$$	$${ } - 2.012^{{***}}$$ $$\left( {0.475} \right)$$
LTCI public	$${ }0.380^{{***}}$$ $$\left( {0.113} \right)$$	$${ } - 0.433^{{***}}$$ $$\left( {0.099} \right)$$	$$0.373^{{**}}$$ $$\left( {0.157} \right)$$	$${ } - 0.243^{{**}}$$ $$\left( {0.121} \right)$$	$${ }0.423^{{***}}$$ $$\left( {0.114} \right)$$	$${ } - 0.453^{{***}}$$ $$\left( {0.102} \right)$$
LTCI private	$0.153$ $\left( {0.358} \right)$	$- 0.192$ $\left( {0.249} \right)$	$- 3.449$ $\left( {100.738} \right)$	$- 0.152$ $\left( {0.315} \right)$	$0.239$ $\left( {0.361} \right)$	$- 0.238$ $\left( {0.270} \right)$
Controls (excl. wealth)^a^	Yes	Yes	Yes	Yes	Yes	Yes
Regional dummies^b^	Yes	Yes	Yes	Yes	Yes	Yes
Pseudo *R*^2c^	14.57%	22.97%	23.76%	27.70%	15.12%	22.91%
*N*	1903	2′029	1903	2029	1903	2029

Dependent variables in the first row

Robust standard errors are reported in parentheses. The significance levels of the two-tailed hypothesis test are coded as follows: * significance at 10% level, ** significance at 5% level, *** significance at 1% level

^a^Control variables used in Section “Empirical results” excluding wealth

^b^Regional binary variables based on the NUTS 2 classification (Regions for Italy and Autonomous Communities for Spain)

^c^Mc. Fadden’s pseudo *R*^2^

The inclusion of regional fixed effects does not substantially change the results reported in Table 5. The coefficients corresponding to public LTC coverage keep the same sign and increase their significance, despite that the sample is slightly reduced due to missing observations (missing regional information). The coefficients corresponding to the effect of private LTC insurance ownership keep the same sign but are non-significant at the usual confidence levels in all models. Finally, we also find that regional fixed effects are important determinants of informal care supply in both samples as pseudo $R^{2}$ are larger in all regressions.

### Eligibility criteria

As a way to make sure that those who declare in the survey to have LTC coverage, either public or private, receive indeed an indemnity, we decide in the following to only consider those individuals who are strongly dependent and declare to have two or more ADL limitations. By considering the subsamples for Italy and Spain, we focus on those individuals who are most likely to be eligible for LTC benefits since by definition of the eligibility criteria only those with a high degree of dependency are eligible for LTC benefits. Our choice of the criterion having two or more ADL limitations is based on the Spanish public LTC benefits eligibility rule as laid out in Section “LTC financing in Italy and Spain”. This criterion is also in line with the practice of other European countries having a public LTC insurance scheme such as France [42] and Germany [43].

Table 8 shows the results of the different regressions with the selected subsamples. From the model specification, we removed the formal care, hospital, health and demographic control variables as well as wealth following the Bayesian information criterion (BIC)^4^ [41], with the objective of maximizing the degrees of freedom of this second econometric estimation given the reduced number of observations in the subsamples.

**Table 8:** Probit regression models on a subsample of individuals with two or more ADL limitations

	Informal care (${\text{IC}}^{1}$)		Informal care ADL (${\text{IC}}^{2}$)		Informal care IADL $\left( {{\text{IC}}^{3} } \right)$		Informal care (${\text{IC}}^{1}$)
	Italy	Spain	Italy	Spain	Italy	Spain	Pooled sample
(Intercept)	$$- 0.912^{{***}} \left( {0.256} \right)$$	$$- 0.689^{{***}} \left( {0.248} \right)$$	$$- 1.199^{{***}} \left( {0.272} \right)$$	$$- 0.978^{{***}} \left( {0.257} \right)$$	$$- 1.075^{{***}} \left( {0.266} \right)$$	$$- 0.748^{{***}} \left( {0.251} \right)$$	$$- 0.679^{{***}} \left( {0.188} \right)$$
LTCI public	$$0.725^{{***}} \left( {0.261} \right)$$	$$- 0.706^{{***}} \left( {0.168} \right)$$	$$0.665^{{**}} \left( {0.266} \right)$$	$$- 0.584^{{***}} \left( {0.174} \right)$$	$${ }0.731^{{***}} \left( {0.263} \right)$$	$$- 0.756^{{***}} \left( {0.173} \right)$$	$$- 0.613^{{***}} \left( {0.163} \right)$$
LTCI private	–	$$- 0.613$$$$\left( {0.460} \right)$$	–	$$- 0.396$$ $$\left( {0.457} \right)$$	–	$$- 0.537\left( {0.462} \right)$$	–
IADL limitations	$$0.072^{**} \left( {0.028} \right)$$	$$0.096^{***} \left( {0.025} \right)$$	$$0.082^{***} \left( {0.030} \right)$$	$$0.103^{***} \left( {0.026} \right)$$	$$0.068^{**} \left( {0.029} \right)$$	$$0.102^{***} \left( {0.026} \right)$$	$$0.086^{***} \left( {0.018} \right)$$
HH members							
2	$$- 0.550^{***} \left( {0.206} \right)$$	$$- 0.507^{***} \left( {0.207} \right)$$	$$- 0.533^{**} \left( {0.212} \right)$$	$$- 0.497^{**} \left( {0.207} \right)$$	$$- 0.554^{***} \left( {0.209} \right)$$	$$- 0.613^{***} \left( {0.208} \right)$$	$$- 0.532^{***} \left( {0.144} \right)$$
3	$$- 0.914^{***} \left( {0.277} \right)$$	$$- 0.958^{***} \left( {0.257} \right)$$	$$- 0.991^{***} \left( {0.298} \right)$$	$$- 0.929^{***} \left( {0.265} \right)$$	$$- 1.040^{***} \left( {0.293} \right)$$	$$- 0.901^{***} \left( {0.257} \right)$$	$$- 0.959^{***} \left( {0.186} \right)$$
4 or more	$$- 1.325^{***} \left( {0.388} \right)$$	$$- 1.539^{***} \left( {0.363} \right)$$	$$- 1.132^{***} \left( {0.391} \right)$$	$$- 1.252^{***} \left( {0.360} \right)$$	$$- 1.268^{***} \left( {0.391} \right)$$	$$- 1.483^{**} \left( {0.363} \right)$$	$$- 1.495^{**} \left( {0.263} \right)$$
*N* children	$$0.081\left( {0.064} \right)$$	$$0.091^{**} \left( {0.039} \right)$$	$$0.124\left( {0.066} \right)$$	$$0.093^{**} \left( {0.039} \right)$$	$$0.086\left( {0.065} \right)$$	$$0.091^{**} \left( {0.039} \right)$$	$$0.097^{***} \left( {0.033} \right)$$
Has daughter	$$0.371^{*} \left( {0.216} \right)$$	$$0.533^{***} \left( {0.189} \right)$$	$$0.262\left( {0.227} \right)$$	$$0.437^{***} \left( {0.197} \right)$$	$$0.509^{**} \left( {0.226} \right)$$	$$0.509^{***} \left( {0.193} \right)$$	$$0.458^{***} \left( {0.139} \right)$$
Catalan	–	$$- 0.571^{**} \left( {0.248} \right)$$	–	$$- 0.503^{**} \left( {0.262} \right)$$	–	$$- 0.569^{**} \left( {0.253} \right)$$	–
Italy	–	–	–	–	–	–	$$- 0.398^{{***}} \left( {0.310} \right)$$
Italy * LTCI public	–	–	–	–	–	–	$$1.363^{***} \left( {0.310} \right)$$
Pseudo *R*^2a^	11.55%–	17.45%	12.02%	15.00%	12.81%	16.97%	14.26%
*N*	267	368	267	368	267	368	635

Robust standard errors are reported in parentheses

^*^Significance at 10% level, ^**^significance at 5% level, ^***^significance at 1% level

^a^Mc. Fadden’s pseudo *R*^2^

The results of this second set of regressions confirm the findings of the previous section. In Italy, we observe a significantly positive effect of public LTC coverage on informal care across all three categories of informal care while in Spain, this relationship is consistently negative. Regarding private insurance, we obtain a non-significant negative effect on the probability of receiving informal care in Spain. Note that in Italy, we could not include this variable in the regression analysis as no individuals declaring to own private LTC insurance remained in the subsample. In the regression with the pooled sample, the effects of public LTCI and the interaction are maintained and the country dummy is negative and highly significant, showing that the probability of receiving care for this subgroup of population is higher in Spain.

In addition, we find that the marginal effects of public LTC coverage on the probability of receiving informal care and their significance levels are larger than in the previous section in both countries despite the sharp reduction in the sample size. Regarding the control variables, with the exception of the number of IADL limitations, coefficients are larger in absolute value but no changes in their sign are observed.

### Bootstrapping the empirical coefficients’ distribution

We further control the robustness of our results using the bootstrap method. The bootstrapping technique, pioneered by Efron [44], consists of a Monte-Carlo simulation randomly drawing a large number of samples from the original set of observations, running the regression model and computing the distribution statistics of the obtained regression coefficients. This makes it possible to estimate the empirical distribution of a given estimator (or set of estimators) with the objective of checking the robustness of the analytical approximation of its values and confidence intervals. Since we work with a non-linear model (i.e. a probit model) and relatively small sample sizes, our context is propitious for the use of such resampling techniques [45].

For each country, we start by selecting 5000 random samples from the initial data. The number of observations in each of these samples corresponds to the size of the original dataset, i.e. 2181 observations for Italy and 2344 for Spain. Note that in random sampling, a same individual can be selected twice (i.e. sampling with replacement). Using the generated samples, the different probit models defined in Section “Empirical results” are estimated. As in Section “Controlling for regional fixed effects”, we do not estimate the model that includes wealth. This way, we obtain 5000 sets of the bootstrapped estimates. From these estimates, it is straightforward to extract their expected values and confidence intervals.

In Table 9, we first present the results of the bootstrapping for the parameters defining LTC coverage ownership in Italy. We provide the simulated coefficients’ two-tailed 95% confidence intervals and mean values.

**Table 9:** The 95% confidence intervals and expected values of the LTCI public and private parameters in Italy

	Informal care (${\text{IC}}^{1}$)	Informal care ADL (${\text{IC}}^{2}$)	Informal care IADL (${\text{IC}}^{3}$)
LTCI public	[0.045|0.406] 0.233	[0.035|0.534] 0.290	[0.073|0.435] 0.258
LTCI private	[– 0.274|0.856] 0.370	[– 3.863|0.874] 1.080	[– 0.217|0.925] 0.435

In Italy, we observe that the 95%-confidence intervals of the parameters corresponding to public LTC coverage do not include zero (no sign change) which allows us to conclude on the robustness of the positive effect of public LTC financing on informal care. Moreover, the expected values of public LTC parameters are very close to the parameter estimates presented in Table 5. Therefore, the hypothesis that public support crowds out informal care is still rejected for Italy in the case of public LTC financing.

The bounds of the confidence intervals surrounding the estimates corresponding to private LTC insurance are of opposite signs making it impossible to judge on the trend of the marginal effect. Thus, no valid conclusions can be extracted for this parameter following the bootstrap exercise. Table 10 presents the results of the bootstrapping for the parameters defining LTC insurance ownership in Spain. In the case of the coefficients corresponding to public LTC coverage in Spain, the 95%-confidence intervals are below zero for all types of informal care, underlining the consistently negative sign of these estimates. Additionally, as in the Italian case, no relevant differences between the simulated expected values and the parameters reported in Section “Econometric analysis” are noticed and in any of these three cases. Therefore, the bootstrap results support the hypothesis that public support decreases informal care in the Spanish public LTC system.

**Table 10:** The 95% confidence intervals and expected values of the LTCI public and private parameters in Spain

	Informal care (${\text{IC}}^{1}$)	Informal care ADL (${\text{IC}}^{2}$)	Informal care IADL (${\text{IC}}^{3}$)
LTCI public	[– 0.621|0.276] 0.443	[– 0.436|0.019] 0.222	[– 0.627|0.272] 0.444
LTCI private	[– 0.913|0.075] 0.370	[– 1.002|0.396] 0.211	[– 0.997|0.004] 0.442

The 95% confidence intervals of the coefficients corresponding to private LTC insurance contain both, negative and positive values, and thus no valid conclusions for these marginal effects can be extracted.

## Conclusion

This article uses cross-sectional data from the sixth wave of the SHARE survey to test the effect of both LTC public benefits and private insurance on the receipt of informal care by non-co-resident family members in Italy and Spain.

The choice of Italy and Spain comes from the fact that informal care is rather similar in these two countries while their respective public LTC financing systems are rather different. Indeed, on one hand, these two Southern European countries are considered as “strong family ties countries” and, therefore, are rather similar in terms of family values with family members representing the main source of informal care. On the other hand, the nature of public benefits is very different, proportional to formal care expenses in Spain, and mainly in the form of cash benefits independent of formal care expenditures in Italy.

We consider three categories of informal care and dissociate informal care for ADL from informal care for IADL, as these two kinds of care can be provided for different reasons and then be influenced to a different extend by insurance.

Our results support the hypothesis of LTC public coverage decreasing informal care for Spain. However, for Italy, we find a consistent positive and significant relationship between LTC public coverage and the probability of receiving informal care by non-co-resident family members. Regarding the effect of private LTC insurance on informal care, we also find significant opposite results for the two countries except when informal care is defined as informal care for ADL. In that case, private LTC insurance positively impacts informal care in Italy and negatively in Spain.

These results tend to confirm that the effect of public benefits on informal care is influenced by the typology of public LTC coverage. In Spain, benefits, either in kind or financial, depend on the consumption of formal care. Hence, benefits are received only if formal care is consumed. The use of formal care is, therefore, encouraged, thus providing much less incentives to offer informal care.

In Italy, public benefits are mainly in the form of cash benefits independent of formal care expenses, complemented by additional regional and municipal LTC services. Therefore, LTC benefits are not necessarily linked to formal care consumption as it happens in Spain. The positive relationship between LTC public coverage and informal care in Italy seems to be explained by the fact that cash benefits can be used to directly provide financial compensation or incentives to informal caregivers. Hence, such cash benefits fulfil their initial role which was thought as a measure to support informal caregivers [23].

Our results also show that in both countries, private LTC insurance, whose benefits are cash and fixed, seems to complement the public LTC financing system in place. This could explain why the direction of the marginal effect of private insurance on informal care follows the one of public LTC coverage and the positive sign of the interaction term in Section “Joint regressions”. A deeper analysis on the potential complementary role of private LTC insurance with public LTC programs can be an interesting topic for future research.

There are several limitations to this study that need to be pointed out. First, our results apply to informal care provided by family members living outside the household and not to informal care received by co-resident and other caregivers such as neighbours and friends. Yet, we focus on the most common type of informal care according to our data and including alternative forms of informal care in the dependent variable would come at the cost of an increase in the main estimates’ heterogeneity, making them more difficult to interpret. The second limitation concerns the fact that LTC benefits in Italy are restricted to severely dependent individuals while in Spain, they can cover those moderately limited as well. While we partially control for this difference, the degree of needs differently impacts eligibility criteria in Spain and Italy, and could influence the relationship between informal care and LTC coverage. A third limitation is that children altruism could justify the positive relationship between LTC coverage and informal care found in Italy as it increases the marginal benefit of supplying care and even to a higher extent in the presence of LTC coverage (Bascans et al. [16], Klimaviciute [13]). However, we are unable to control for this phenomenon with our data.

To conclude, whether a LTC system is more or less prone to influence, positively or negatively, informal care might lead to different economic policies. According to our results, a model similar to the Italian public system mainly based on fixed benefits would provide few disincentives, even any, to informal care givers. This would help to attenuate LTC expenditures’ increases. While, a model similar to the Spanish public system with proportional benefits provides disincentives to informal caregivers and then could be socially beneficial by reducing the burden of caregiving in terms of health and lower employment participation. Further research on these issues and for other countries should be developed to generalise our results.

## Footnotes

1 LTC is a mix of social and health care provided on a daily basis, formally or informally, at home or in institutions, to people suffering from a loss of autonomy in their daily living activities.

2 Exceptionally, these services can also be offered by family members living in the same household as the dependent elderly [30].

3 The original question in SHARE reads as “Is there someone living in this household who has helped you regularly during the last twelve months with personal care, such as washing, getting out of bed, or dressing?”.

4 The BIC is used as, compared to the AIC, it penalizes more those models with a large number of parameters.
